# Double Burden of Malnutrition and Inequalities in the Nutritional Status of Adults: A Population-Based Study in Brazil, 2019

**DOI:** 10.3389/ijph.2021.609179

**Published:** 2021-05-28

**Authors:** Fernanda Oliveira Meller, Antônio Augusto Schäfer, Leonardo Pozza Santos, Micaela Rabelo Quadra, Vanessa Iribarrem Avena Miranda

**Affiliations:** ^1^ Postgraduate Program in Public Health, University of Southern Santa Catarina, Criciúma, Brazil; ^2^ Nutrition College, Federal University of Pampa, Itaqui, Brazil

**Keywords:** health inequalites, underweight, overweigh, nutritional status, adult

## Abstract

**Objective:** To describe the inequalities in the double burden of malnutrition (DBM) in the adult population.

**Methods:** Study carried out with data from the VIGITEL study, conducted in 2019 in all Brazilian capitals. Underweight and excess weight were evaluated on the basis of years of schooling and age. Multi-level analysis was performed including Human Development Index of each capital and individual-level variables. The inequality slope index was used to assess the magnitude of the inequalities found. All analyses considered the svy command owing to the complexity of the sampling process.

**Results:** 47.119 individuals were studied. Men with no education had 6 percentage points more underweight compared to those with higher education. Higher prevalence of excess weight was found among men with higher education and women with no education. In women, the difference was 18 percentage points between extreme categories. Elderly people with no education had 10 percentage points more excess weight than those with higher education.

**Conclusions:** The findings suggest the need for intersectoral actions that can cope with the social inequalities and help confronting with the DBM in Brazil.

## Introduction

Nutritional status can indicate associations between health conditions and social and economic context of population groups, as well as predict adverse outcomes throughout life [1, 2]. In the last few years, the expression “double burden of malnutrition (DBM)” has been highlighted in scientific publications for addressing an existing situation linked to the fast and intense process of epidemiological, nutritional and demographic transitions, especially in low-income and middle-income countries: the coexistence of malnutrition and excess weight [3].

In epidemiological studies, nutritional status has been mostly identified through the Body Mass Index (BMI), because this measure is easy and fast to apply, and it allows classifying individuals into underweight, normal weight, overweight and obesity [1]. The BMI is considered an extremely important tool for identification of overall body fat and all its associated factors, since it presents high correlation with total adiposity, although its capacity of predicting body fat distribution is very controversial [4, 5].

Excess weight is defined as body fat accumulation [4], which can be influenced by sociodemographic, behavioral and genetic factors [6]. The metabolic disorders arising from overweight and obesity are capable of causing inflammatory processes, oxidative stress and nutritional disorders [6], leading to several consequences, such as the development of non-communicable chronic diseases (NCDs) and early mortality [7–9]. Worldwide, about 1.9 billion people were overweight or obese in 2016 [7]. In Brazil, in the last 13 years, there was an increase of 12.4% in the prevalence of excess weight in the adult population [10, 11].

Conversely, malnutrition is frequently characterized by weight or height deficit [12]. It is more prevalent in children, but it also affects adults and, specially, the elderly people, whose proportion in total population has increased as a result of the demographic transition process [12, 13]. Between 2017 and 2019, the worldwide prevalence of underweight, characterized by a BMI <18.5 kg/m^2^, in adults was below 10%, and less than 2.5% of the Brazilian population was affected by this condition [14]. On the other hand, the prevalence of underweight is higher in Brazilian elderly (19.9% in men and 18.2% in women in 2009) [15], which can be caused by the progressive loss of muscle mass inherent to the aging process [16]. Social inequality, lower level of education and lower income are three of the most important factors associated with underweight in all age groups [17].

It is known that characteristics such as geographic location, age, sex, ethnicity, socioeconomic status and educational level can increase the risk of nutritional disorders like underweight or excess weight [18]. Important studies support this assumption, with evidence that education, income and place of residence are related to both underweight and excess weight in adult populations [17, 19].

In addition, Popkin et al. [3] emphasized the fact that the scientific community needs to be more aware of the DBM, especially in low-income and middle-income countries, such as Brazil, where a coexistence of underweight, excess weight and NCDs is observed, sometimes even in a same population stratum. Most nationally representative studies have given more attention to overweight and obesity, and placed little emphasis on underweight in the adult and elderly population of Brazil. Therefore, the present study aimed to describe the coexistence of underweight and excess weight in the Brazilian adult population according to age and years of schooling, as well as the socioeconomic and demographic inequalities associated with these nutritional disorders.

## Methods

This is a cross-sectional study using data from the Surveillance System of Risk and Protective Factors for Chronic Diseases by Telephone Survey (“*Vigitel*” in the Brazilian acronym), conducted in 2019 in the 26 Brazilian capital states and Federal District [11]. Vigitel is a population-based survey with adults (aged 18 or over) who live in the capitals or in the Federal District, whose aim is to monitor the frequency and distribution of the main determinants of NCDs in the Brazilian population.

Vigitel has been conducted annually since 2006, and the sampling process takes place in two stages and aims to obtain probabilistic samples from the population of adults living in households with at least one landline telephone number. In the first stage, residential landlines indexed to the electronic database of telephone companies are randomly selected in each city. In the second stage, one of the adults residing in the selected household is randomly selected to participate in the study answering the questionnaire. At the end of 2019 Vigitel, 52,443 interviews were conducted. Further details on the Vigitel survey methodology can be found in the final published report [11]. Vigitel databases are publicly available and can be assessed at http://svs.aids.gov.br/download/Vigitel.

### Nutritional Status (Underweight and Excess Weight)

The study outcomes were underweight and excess weight assessed using BMI, determined by dividing weight (in kilograms) by height (in meters) squared. Both measures of weight and height were self-reported and collected in the telephone interviews. The participants’ nutritional status was classified according to the BMI cut-offs recommended by the World Health Organization (WHO) [1]. Young adults (under 20 years of age) and adults (20–59 years of age) whose BMI was <18.5 kg/m^2^ were classified as underweight. On the other hand, those young adults and adults with BMI ≥25 kg/m^2^ were classified with excess weight [1, 20]. Despite differences in nutritional status classification among young adults and adults, we have chosen the same cut-off point to classify underweight and excess weight of both groups because the cutoff points of the 2007 World Health Organization growth standards, used to classify nutritional status of adolescents, are very similar to ones used for adults from the 18 years of age [21]. Nutritional status of individuals with 60 years of age or older were classified using the specific cutoff points proposed by Lipshitz et al. [22] and recommended by the Brazilian Ministry of Health. According to this classification, those individuals with BMI <22 kg/m^2^ were classified as underweight, while those elderly with BMI ≥27 kg/m^2^ were classified with excess weight [23]. We have chosen the specific cutoff points for elderly people as this classification seems to be more adequate to predict mortality in this population [24].

### Years of Schooling and Age

We included years of schooling and age as the main exposures of our study because these two characteristics are important predictors of nutritional status. Since household income is not investigated in Vigitel, we used years of schooling as a proxy of socioeconomic status. In the 2019 Vigitel, years of schooling was collected in completed years of formal education. We categorized years of schooling in five groups, considering the different levels of Brazilian education system (No education, 1 to 4, 5 to 8, 9 to 11 and 12 years or more). Age was collected as completed years and categorized in six groups, as follow: <20 years, 20 to 29, 30 to 39, 40 to 49, 50 to 59 and 60 years or more.

### Other Socioeconomic and Demographic Characteristics

Information on other socioeconomic and demographic characteristics potentially associated with both outcomes and exposures were included as potential confounders of our study. As socioeconomic factor, we included the Human Development Index (HDI) of each capital for the year 2010 (the last HDI available information for all Brazilian capitals). HDI information was gathered from the Brazilian Institute of Geography and Statistics. We also included the following individual-level variables collected in the 2019 Vigitel: geographical region (categorized according to the five regions of Brazil - North, Northeast, Mid-west, Southeast and South), marital status (categorized in married or not married), self-reported skin color (white, brown and black) and household size, defined according to the number of members in the household (1 or 2, 3 or 4 and 5 or more members).

### Statistical Analyses

Differences in nutritional status (underweight and excess weight) according to years of schooling and age were assessed using Pearson’s Chi-square tests for heterogeneity or linear tendency and Fisher's Exact test, using a 5% significance level and 95% confidence intervals.

After that, adjusted Poisson regression was used to check whether crude differences in underweight and excess weight according to years of schooling and age were independent of other socioeconomic and demographic factors. Adjusted analyses were conducted in two main steps. In the first step, we adjusted all models for individual-level variables to address whether significant differences observed in the crude model were independent of individual socioeconomic and demographic factors. The individual-level variables included as potential confounders were geographical region, marital status, skin color and household size, all of them associated with, at least, one outcome and one exposure at 20% significance level. The second step consisted in a multilevel analysis where the prevalence ratio of nutritional status per education and age was estimated using multilevel Poisson models, adjusted for individual-level characteristics plus HDI, the cities-level variable included in the model.

Finally, socioeconomic and demographic inequalities were further analyzed using a formal test to assess inequality. For this purpose, we used the *Slope index of inequality* (SII) which represents absolute measures of inequality. The SII is obtained through linear regression between the outcome and the independent variable, inserted in the model as an ordinal variable [25]. The index refers to the difference between the extreme groups of the independent variables used in the analysis of inequality. Equiplots were created to illustrate the observed inequalities.

All analyses were conducted in the Statistical package Stata, version 16.1 and were stratified by sex. In addition, all prevalence rates were estimated using weighting factors (command ‘svy’ in Stata), considering the complex sampling of Vigitel.

## Results

Of the 52,443 interviewed individuals in the 2019 Vigitel, 52,349 had available information for BMI and were included in the analysis, after exclusion of outliers. They were more likely to be female (54.0%) and more likely to live in Southeast region (45%). Regarding marital status, more than a half of the interviewed individuals was not married. Additionally, the majority of the sample was composed by brown or black people and more than one quarter reported to live in households with five or more members. Mean age was 42.7 years, and subjects aged 60 years or older represented a little more than 20% of the sample. Finally, more than one third of the sample reported 12 or more years of schooling (Table 1).

**TABLE 1:** Sociodemographic description and nutritional status of the sample. VIGITEL, 2019 (52,443).

	Male	Female	Total
	% (95% CI)	% (95% CI)	% (95% CI)
Geographical region			
North	10.8 (10.1; 11.4)	10.0 (9.6; 10.5)	10.4 (10.0; 10.7)
Northeast	24.8 (23.8; 25.9)	25.6 (24.8; 26.4)	25.2 (24.6; 25.9)
Mid-west	12.0 (11.1; 12.8)	11.6 (11.0; 12.1)	11.8 (11.3; 12.3)
Southeast	44.4 (42.8; 46.1)	44.8 (43.6; 46.0)	44.6 (43.6; 45.6)
South	8.0 (7.5; 8.6)	8.0 (7.6; 8.4)	8.0 (7.7; 8.4)
Marital status			
Married	48.5 (47.0; 50.1)	44.6 (43.5; 45.8)	53.6 (52.6; 54.5)
Not married	51.5 (50.0; 53.0)	55.4 (54.2; 56.5)	46.4 (45.5; 47.4)
Skin color			
White	42.3 (40.7; 43.9)	46.8 (45.6; 48.1)	44.8 (43.8; 45.8)
Brown	44.5 (42.9; 46.1)	42.9 (41.7; 44.1)	43.6 (42.6; 44.6)
Black	13.2 (12.1; 14.4)	10.3 (9.5; 11.0)	11.6 (11.0; 12.3)
Household size (number of members)			
1–2	17.3 (16.4; 18.2)	21.9 (21.2; 22.7)	19.8 (19.2; 20.4)
3–4	55.3 (53.8; 56.9)	50.6 (49.4; 51.7)	52.8 (51.8; 53.7)
5 or more	27.3 (25.8; 28.9)	27.5 (26.3; 28.7)	27.4 (26.5; 28.4)
Age groups			
18–19	5.0 (4.4; 5.7)	3.5 (3.0; 3.9)	4.2 (3.8; 4.6)
20–29	26.7 (25.2; 28.2)	19.0 (18.0; 20.1)	22.5 (21.6; 23.5)
30–39	20.3 (19.0; 21.7)	20.5 (19.5; 21.6)	20.4 (19.6; 21.3)
40–49	17.3 (16.2; 18.5)	19.3 (18.4; 20.3)	18.4 (17.7; 19.1)
50–59	15.3 (14.4; 16.3)	16.9 (16.2; 17.8)	16.2 (15.6; 16.8)
60 or more	15.4 (14.6; 16.3)	20.8 (20.0; 21.5)	18.3 (17.8; 18.8)
Years of schooling			
0	1.8 (1.4; 2.1)	2.4 (2.1; 2.7)	2.1 (1.8; 2.3)
1 to 4	10.5 (9.6; 11.6)	10.8 (10.2; 11.5)	10.7 (10.1; 11.3)
5 to 8	16,6 (15.4; 17.9)	15.5 (14.7; 16.4)	16.0 (15.3; 16.8)
9 to 11	39.9 (38.4; 41.4)	37.1 (36.0; 38.2)	38.4 (37.5; 39.3)
12 or more	31.2 (29.8; 32.6)	34.2 (33.1; 35.4)	32.8 (31.9; 33.7)
Total	**46.0 (45.0; 46.9)**	**54.0 (53.1; 55.0)**	**100.0**

95% CI–95% Confidence interval. Highest missing values for self-reported skin color (5,522).

The estimated prevalence of underweight was higher in women when compared to men (6.1% vs. 3.9%). When evaluating underweight prevalence according to the age groups analyzed, we found that underweight prevalence was relatively low for adults (20–59 years old), but much higher in the extreme age groups: around 10% in male and female young adults, and over 12% among the elderly population. It is interesting to notice that underweight prevalence was always higher in women, independent of the age strata (Table 2). The relationship between underweight prevalence and years of schooling evidenced an inverse association for both sexes: while underweight prevalence affected around 15% of both men and women with no formal education, less than 2% of men and 5% of women with 12 or more years of schooling were classified as underweight. Again, underweight prevalence was higher in women in almost all education strata when compared to men (Table 2).

**TABLE 2:** Crude prevalence of underweight and excess weight according to age groups and education, stratified by sex. VIGITEL, 2019 (52,443).

	Underweight^a^		Excess weight^b^	
	Male % (95% CI)	Female % (95% CI)	Male % (95% CI)	Female % (95% CI)
Age groups	<0.001	<0.001	<0.001	<0.001
18–19	8.9 (5.6; 13.9)	11.2 (7.8; 15.7)	26.4 (20.8; 33.0)	26.5 (21.1; 32.6)
20–29	3.4 (2.4; 4.6)	6.5 (5.1; 8.3)	43.2 (39.8; 46.7)	37.6 (34.4; 40.8)
30–39	1.1 (0.6; 2.0)	3.2 (2.2; 4.7)	65.3 (61.5; 69.0)	54.9 (51.9; 58.0)
40–49	1.3 (0.6; 2.5)	1.8 (1.3; 2.5)	66.5 (63.0; 69.8)	58.6 (56.1; 61.1)
50–59	1.8 (0.8; 3.9)	2.2 (1.6; 3.0)	65.1 (61.7; 68.3)	61.5 (59.1; 63.8)
60 or more	12.1 (10.5; 13.9)	14.8 (13.8; 16.0)	42.2 (39.6; 44.7)	44.1 (42.4; 45.7)
Years of schooling	<0.001	<0.001	0.003	<0.001
0	13.4 (7.0; 24.1)	16.3 (12.0; 21.8)	50.0 (40.3; 59.7)	49.2 (42.3; 56.2)
1 to 4	7.2 (5.5; 9.5)	10.2 (8.7; 11.9)	53.4 (48.3; 58.3)	51.4 (48.3; 54.5)
5 to 8	5.2 (3.8; 7.2)	5.4 (4.5; 6.3)	54.3 (50.1; 58.5)	58.7 (55.6; 61.7)
9 to 11	3.7 (2.9; 4.6)	5.5 (4.7; 6.5)	51.0 (48.6; 53.4)	52.4 (50.5; 54.3)
12 or more	1.9 (1.4; 2.6)	5.1 (4.3; 6.0)	58.4 (55.6; 61.0)	43.6 (41.6; 45.7)
Total	**3.9 (3.4; 4.5)**	**6.1 (5.6; 6.6)**	**54.1 (52.5; 55.6)**	**50.2 (49.0; 51.4)**

^a^ Body mass index <18.5 kg/m^2^ for adults and <22 kg/m^2^ for elderly.

^b^ Body mass index ≥25 kg/m^2^ for adults and ≥27 kg/m^2^ for elderly. Displayed *p*-values from Wald test.

95% CI–95% Confidence interval.

Regarding to excess weight, men presented higher prevalence than women (54.1% vs. 50.2%), although more than a half of the sample have presented BMI >25 kg/m^2^ in both sexes. When we assessed excess weight prevalence according to age groups, higher prevalence rates were observed for individuals aged 30–59 years, and in men aged 30–59 years these prevalence rates were higher than 60%. On the other hand, lower prevalence values were observed in the extreme age groups: a little more than 25% of young adults presented excess weight, while a little more than 40% of the elderly sample presented BMI >27 kg/m^2^ (Table 2). Relationship between excess weight and years of schooling stratified by sex evidenced an interesting result: a direct association in men, indicating higher prevalence of excess weight in the more educated group, but an inverse association in women, demonstrating a lower prevalence of excess weight in the more educated ones (Table 2).

Adjustment for socioeconomic and demographic factors included as potential confounders of our study evidenced no changes in the observed associations. The fully adjusted model (multi-level analysis) showed that underweight prevalence remained higher in women, in the extreme age groups, and in the less educated people, independent of capital’s HDI as well as individual’s geographical region, marital status, skin color and household size. Oppositely, excess weight prevalence remained lower in the extreme age groups, higher in the more educated men but lower in the more educated women, independent of the potential confounders included in analyses. It is important to highlight that despite differences in excess weight prevalence according to age and education, the levels remained high in all strata analyzed here (Table 3).

**TABLE 3:** Adjusted prevalence of underweight and excess weight according to age groups and education, stratified by sex. VIGITEL, 2019 (52,443).

	Underweight^a^				Excess weight^b^			
	Adjusted model^c^		Fully adjusted model^d^		Adjusted model^c^		Fully adjusted model^d^	
	Male % (95% CI)	Female % (95% CI)	Male % (95% CI)	Female % (95% CI)	Male % (95% CI)	Female % (95% CI)	Male % (95% CI)	Female % (95% CI)
Age groups	<0.001	<0.001	<0.001	<0.001	<0.001	<0.001	<0.001	<0.001
18–19	5.6 (2.8; 8.3)	9.4 (6.1; 12.7)	8.0 (7.8; 8.1)	11.8 (11.7; 12.0)	30.8 (23.5; 38.2)	26.9 (20.7; 33.1)	26.2 (26.0; 26.3)	27.3 (27.1; 27.4)
20–29	2.6 (1.8; 3.5)	5.7 (4.3; 7.1)	3.4 (3.3; 3.5)	6.8 (6.7; 6.8)	47.2 (43.3; 51.1)	38.0 (34.8; 41.3)	43.5 (43.4; 43.7)	37.4 (37.3; 37.5)
30–39	1.1 (0,4; 1.5)	2.9 (1.6; 4.1)	1.1 (1.0; 1.1)	3.0 (2.9; 3.1)	65.7 (61.8; 69.7)	54.2 (51.1; 57.4)	66.9 (66.5; 67.3)	54.9 (54.8; 55.0)
40–49	1.0 (0.2; 4.0)	1.6 (1.1; 2.1)	0.8 (0.7; 0.8)	1.6 (1.5; 1.6)	64.9 (61.6; 68.3)	57.7 (55.1; 60.3)	68.8 (68.4; 69.1)	58.7 (58.6; 58.9)
50–59	2.1 (0.2; 4.0)	2.2 (1.4; 3.1)	1.5 (1.4; 1.6)	2.2 (2.1; 2.3)	61.9 (58.5; 65.4)	60.9 (58.4; 63.4)	67.1 (66.8; 67.3)	61.0 (60.9; 61.2)
60 or more	17.7 (14.0; 21.4)	15.1 (13.6; 16.7)	11.3 (11.1; 11.4)	15.4 (15.3; 15.4)	39.3 (36.5; 42.0)	44.9 (43.0; 46.9)	43.6 (43.5; 43.7)	43.3 (43.2; 43.3)
Years of schooling	<0.001	<0.001	<0.001	<0.001	<0.001	<0.001	<0.001	<0.001
0	8.1 (3.9; 12.4)	15.6 (10.0; 21.3)	6.3 (6.1; 6.6)	17.8 (17.4; 18.3)	51.9 (41.2; 62.6)	50.7 (42.2; 59.2)	54.9 (54.0; 55.8)	48.9 (48.5; 49.3)
1 to 4	7.6 (5.1; 10.2)	8.9 (7.4; 10.4)	6.4 (6.3; 6.5)	9.7 (9.6; 9.8)	50.3 (45.2; 55.4)	52.1 (48.6; 55.6)	55.4 (55.0; 55.8)	50.7 (50.5; 50.9)
5 to 8	5.6 (3.5; 7.6)	5.1 (4.1; 6.1)	4.8 (4.7; 4.9)	5.4 (5.3; 5.4)	50.8 (46.6; 55.0)	58.1 (54.9; 61.4)	55.3 (54.9; 55.7)	57.1 (56.9; 57.3)
9 to 11	3.6 (2.8; 4.4)	5.4 (4.5; 6.4)	3.5 (3.4; 3.5)	5.7 (5.6; 5.8)	52.1 (49.7; 54.6)	51.6 (50.0; 53.4)	53.4 53.1; 53.6)	51.4 (51.3; 51.5)
12 or more	1.7 (1.2; 2.3)	4.8 (4.0; 5.7)	1.6 (1.6; 1.7)	5.2 (5.1; 5.2)	61.0 (58.3; 63.7)	44.4 (42.2; 46.5)	62.5 (62.3; 62.8)	43.5 (43.3; 43.6)

^a^ Body mass index <18.5 kg/m^2^ for adults and <22 kg/m^2^ for elderly.

^b^ Body mass index ≥25 kg/m^2^ for adults and ≥27 kg/m^2^ for elderly.

^c^ Adjusted for geographical region, marital status, skin color and household size.

^d^ Adjusted for geographical region, marital status, skin color and household size and capitals’ HDI.

Displayed *p*-values from Wald test.

95% CI–95% Confidence interval.

**FIGURE 1:**
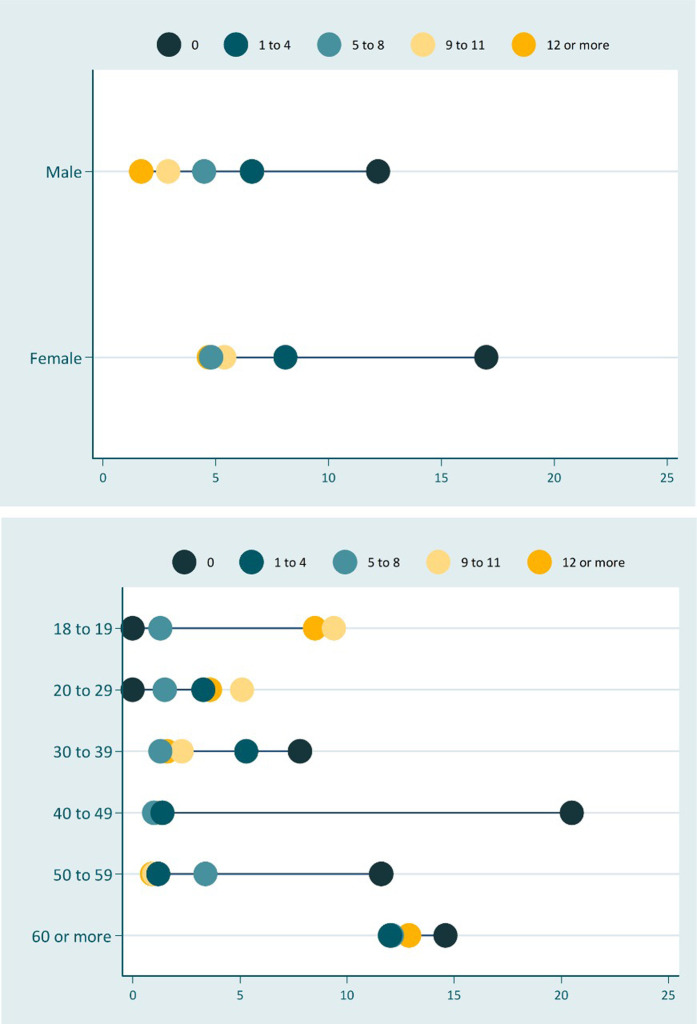
Inequality in underweight according to schooling stratified by sex and age group. Surveillance System of Risk and Protective Factors for Chronic Diseases by Telephone Survey, Brazil, 2019.

**FIGURE 2:**
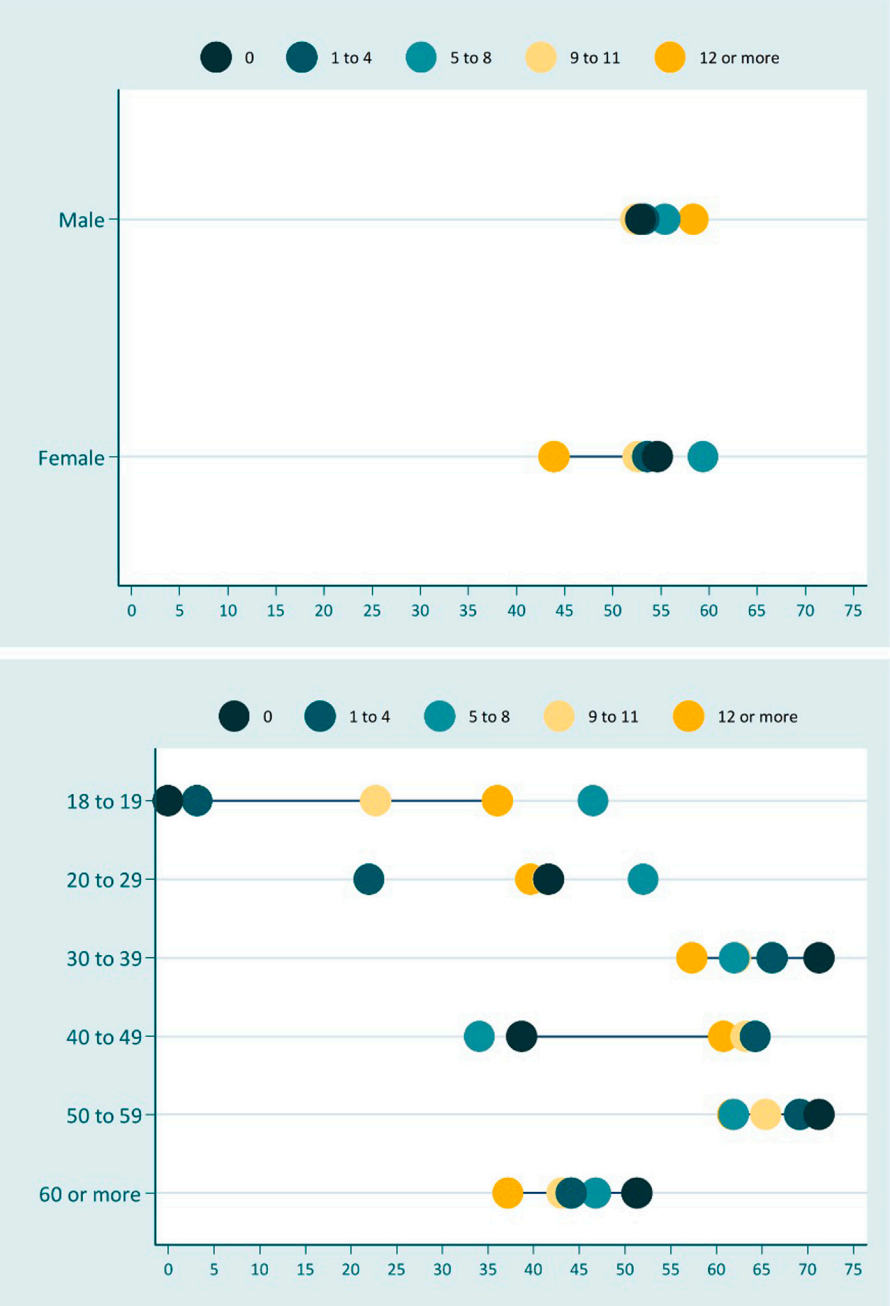
Inequality in excess weight according to schooling stratified by sex and age group. Surveillance System of Risk and Protective Factors for Chronic Diseases by Telephone Survey, Brazil, 2019.

Table 4 shows the absolute inequalities in nutritional status, through the complex inequality index (SII), while Figures 1, 2 present the magnitude of these inequalities. In the general sample, both underweight and excess weight were more concentrated among those individuals with no education. In addition, underweight was more concentrated among uneducated adults of both sexes, with men showing the greatest inequality. Individuals with no schooling had an underweight prevalence 6 percentage points higher when compared to those who studied for at least 12 years.

**TABLE 4:** Absolute inequality (Slope index of inequality) in underweight and excess weight according to schooling stratified by sex and age group. VIGITEL, 2019.

	Underweight^a^		Excess weight^b^	
	ß	*p*-value	ß	*p*-value
Sex				
Male	−6.1	<0.001	6.1	0.041
Female	−3.2	<0.001	−18.4	<0.001
Age group				
18 to 19	4.2	0.358	6.9	0.662
20 to 29	−1.3	0.423	−5.6	0.267
30 to 39	−1.6	0.308	−9.8	0.061
40 to 49	−1.6	0.283	−2.6	0.551
50 to 59	−3.1	0.050	−5.1	0.183
60 or more	0.1	0.356	−9.8	<0.001
Total	−4.3	<0.001	−7.0	<0.001

^a^ Body mass index <18.5 kg/m^2^ for adults and <22 kg/m^2^ for elderly.

^b^ Body mass index ≥25 kg/m^2^ for adults and ≥27 kg/m^2^ for elderly.

Regarding inequality in excess weight, men and women showed an opposite pattern: excess weight was more concentrated among men with higher education and among women without education. The difference was 18 percentage points between the extreme categories of schooling in women and 6 percentage points in men. In addition, elderly people with no education had about 10 percentage points more excess weight than the more educated ones.

## Discussion

Our study, which aimed to describe the prevalence of underweight and excess weight in the adult population living in Brazilian capitals according to age and years of schooling, as well as the socioeconomic and demographic inequalities associated with these conditions, pointed out important results about the DBM in Brazil. Although excess weight had been the most evident problem in the adult Brazilian population, since more than 50% of individuals have presented a BMI above 24.9 kg/m^2^, specific subgroups of the population also have a high prevalence of underweight, as was the case of individuals under 20 and those aged 60 or over.

In recent years, several studies have evidenced that the prevalence of excess weight is at high levels not only in the adult Brazilian population [13, 26], but also in adolescents and children, for whom the increase in overweight and obesity have been faster than in adults [26, 27]. It is known that high prevalence of excess weight is not an exclusive situation for the Brazilian population, since several countries around the world face a similar problem [7, 13, 26].

Although there is no consensus on the role of excess weight in increasing the risk of early mortality, some evidence indicates that any level of overweight could already be responsible for increasing the overall risk of mortality for individuals [3, 4]. In South America, a study carried out using data from the 2014 Vigitel, in Brazil, and the 2013 National Risk Factors Survey, in Argentina, showed a positive association between the prevalence of obesity and overall mortality in both countries at the aggregated level [28].

Nevertheless, our study showed that not only excess weight is a reality in the Brazilian population, but underweight also affects a significative proportion of the population, specially in specific sub-groups. In 2019, only 4% of the adult population in Brazilian capitals presented a BMI<18.5 kg/m^2^. It represents a half of the underweight prevalence in the Brazilian adult population of 30 years ago [29] and about a half of the worldwide prevalence of underweight in 2014 [13]. However, the prevalence of underweight is higher when some specific subgroups of the population are considered. While the prevalence of BMI<18.5 kg/m^2^ was around 2% for adults aged between 20 and 59 years old, this condition was about four times more frequent in those under 20 years old and about six times more frequent in the elderly population.

It is important to take into account that BMI does not distinguish fat mass and fat-free mass, which makes it difficult to accurately analyze the malnutrition situation, mainly in the elderly people. BMI does not allow to identify the progressive loss of muscle mass which is characteristic of aging along with other important factors in the assessment of malnutrition in the elderly. Representatives of the main Clinical Nutrition societies developed a recent consensus recommending that the diagnosis of underweight in adults should consider two important criteria: a phenotypic (weight loss, low BMI or reduced lean mass) and an etiological one (reduced intake of nutrients or ongoing inflammatory process) [30]. As in the present study the diagnosis of underweight considers only a phenotypic factor (low BMI), it is assumed that the prevalence of underweight in the sample of elderly analyzed might be underestimated.

The results of the present study also suggest important disparities in the nutritional status of Brazilian adults. The group of women with less education was the one which the double burden of malnutrition was more severe, since they were more affected by underweight and excess weight. In men, results for underweight and excess weight according to education was opposite ways, i.e., underweight more prevalent in less educated men and excess weight more frequent among the more educated ones.

Previous studies have showed that the socioeconomic status has different influences on the individuals nutritional status [31–33]. A study with data from Vigitel 2006–2009 has showed an increase of obesity in the Brazilian population associated with socioeconomic factors. Less educated women and higher educated men have showed an increase of 1.29–1.34 times and 1.25–1.29 times, respectively, in the prevalence of obesity. Furthermore, unemployed women and employed men also have showed an increase of 1.23 times and 1.24 times, respectively, in the prevalence of obesity [31]. On the other hand, a research with data of the adult Brazilian population has found that the prevalence of overweight and obesity increased in all levels of education between 2006 and 2013, while the increase in the prevalence of BMI >40 kg/m^2^ was more significant in the higher level of education [32]. Another recent review and meta-analysis that aimed to assess the association between socioeconomic status and BMI in adults found a higher risk of overweight in women with low socioeconomic status [33], in line with the findings of our study.

The increase in excess weight can be partly explained by changes in food systems that increased the consumption of ultra-processed foods in the last years. The way people eat, drink, commute to work and pursue leisure activities has also been affecting the distribution of body composition, contributing to the high rates of overweight and obesity worldwide [3]. However, this process, commonly called nutritional transition, has not made the problem of nutritional deficits and low BMI in Brazilian society disappear. From the point of view of public health, it is important to understand the consequences of both underweight and overweight in order to improve the quality of life of the population and avoid early death.

A study carried out with data from the Family Budget Survey, with approximately 56 thousand households, showed that overweight is directly related to the private health expenditure of Brazilian families, which can contribute to increase inequalities, since the less affluent population spends a higher proportion of their income on health when compared to the more affluent families (6% and 3.4%, respectively) [34]. In addition, being overweight brings costs to several other health care sectors. Kent et al. [35], in their systematic review on BMI and health costs, demonstrated that compared to individuals with adequate BMI, health care had an increase in the median annual costs of 12% and 36% for those with overweight and obesity, respectively. According to the authors, these costs were the result of medications, hospitalizations and outpatient care.

Special emphasis should be placed on the National Food and Nutrition Policy (PNAN), due to its importance in providing a basis for food and nutrition initiatives in the Unified Health System, the Brazilian public health system. The purpose of PNAN is to promote adequate and healthy dietary habits, to monitor the nutritional status of the population, as well as prevent nutritional problems [36]. In addition to this important Brazilian policy, there are income transfer programs, which are recognized as tools for reducing poverty and food insecurity, with positive effects on underweight [37]. In Brazil, the Bolsa Família Program is one of the strategies responsible for increasing the availability of food to low-income families, favoring the diversity and quality of their diet [38]. However, despite these actions, inequality still exists, especially in vulnerable groups in which low weight is still a long-standing problem [36].

The most relevant limitation of this study is the nature of the Vigitel sample, composed only by the population with a landline and residing in Brazilian capitals. However, the first factor is attenuated through sample weights, responsible for approximating the study population to the estimated population. Another limitation to be considered is the use of BMI in the assessment of nutritional status; as previously noted, this measure does not distinguish the individuals’ body composition. The strengths of the study are the focus on the DBM in Brazilian capitals, the use of the complex index to assess the magnitude of inequality, and the analyses of intersectionality.

In conclusion, there is a DBM in Brazilian capitals, which is more evident in less-educated women and elderly population. This scenario is also evident in other low-income and middle-income countries. Such a problem requires interconnected actions by different health sectors to prevent and combat both undernutrition and excess weight, because the global objectives of sustainable development include the eradication of hunger and the prevention of malnutrition in all its forms [39]. Therefore, it is of paramount importance to develop actions to face the double burden of malnutrition while taking into account the social disparities found in the Brazilian population.

## Data Availability

Publicly available datasets were analyzed in this study. This data can be found here: http://svs.aids.gov.br/download/Vigitel/.
